# Effect of temperature patterns on iron nugget formation in fluxless processing of titanomagnetite

**DOI:** 10.1038/s41598-022-12968-x

**Published:** 2022-05-27

**Authors:** Zulfiadi Zulhan, Cheryl Livia Sutandar, Indah Suryani, Eddy Agus Basuki

**Affiliations:** Department of Metallurgical Engineering, Faculty of Mining and Petroleum Engineering, Bandung Institute of Technology, Jl. Ganesa No. 10, Bandung, 40132 Indonesia

**Keywords:** Engineering, Chemical engineering

## Abstract

The technology used to process titanomagnetite is currently limited to the rotary kiln-electric furnace. Other techniques are being developed, including the separation of iron in the form of iron nugget from the reduction of titanomagnetite with coal without any flux addition. The effect of different temperature patterns on the formation of iron nuggets from titanomagnetite was studied. The initial temperature was varied from 700 to 1380 °C, while the final temperature was kept constant at 1380 °C. The experiment results showed that the initial temperature affected the formation of iron nuggets. Initial temperatures of 700–1100 °C produced many iron nuggets up to 3 mm in size and an initial temperature of 1200 °C produced one nugget with a size of about 4 mm. Initial temperatures of 1300 and 1380 °C did not produce any iron nuggets due to the formation of metallic iron crust on the surface of the reduced briquettes. The optimum initial temperature was 1000 °C to achieve high iron recovery in the nuggets.

## Introduction

Titanomagnetite (TTM) is one of the raw materials to produce iron, titania, and vanadium. Indonesia is one of the countries that has titanomagnetite resources, mainly from the iron sand on the beach, in the amount of 941 million tons. The technology used to process TTM is currently limited to the rotary kiln-electric furnace^1,2^. It is necessary to add limestone to adjust the chemical composition of the slag for the smelting stage in the electric furnace, which negatively impacts the titania content in the slag. Fluxless smelting of TTM in the electric furnace was suggested to increase the titania content in the slag^3^.

In addition, a blast furnace can be used, but the TTM must be mixed with ordinary iron ore where the maximum amount of TTM is about 65% in the mixture^4,5^. Due to the lower operating temperature of the blast furnace than the electric furnace, it is necessary to add more fluxes which can dilute the titania content in the slag. In addition, the other problems are the formation of TiC and TiN because the atmosphere in the hearth blast furnace is very reductive and the use of hot blast air in the tuyeres as a source of nitrogen. The TiC and TiN cause the slag to become viscous, which causes a lot of metal to be trapped and enter the slag^6^.

Alternatively, many studies have focused on direct reduction in the solid-state of TTM by carbonaceous materials wherein iron can be separated from other oxides using magnetic separators^7–23^. Coal as a reducing agent had shown better performance than coke, graphite, or biochar^21^. The addition of coal can be done simultaneously through mixing with TTM or by immersing pellets or briquettes into the coal bed. The optimum temperature for the TTM reduction was 1200 °C^17,21^. The addition of sodium sulfate, sodium carbonate, and calcium fluorite as additives were also investigated to promote iron migration, accumulation, and growth into larger particles^10,13,16,18,23^. The nugget formation with a treatment time of 440 min and temperatures up to 1350 °C was observed by Hu et al.^8^, but in the experiments using a laboratory-scale rotary hearth furnace at a temperature of 900—1350 °C which was divided into three zones, no nuggets formation was reported^12^. The formation of iron nuggets from primary iron ore, namely hematite, was investigated by Matsumura et al.^24^ in 1996 which later became the basis for the development of ITMk3 technology by Kobe Steel^25–27^.

In previous work, we have introduced that iron nuggets were formed by reduction of TTM/coal composite pellets using an isothermal-temperature gradient pattern where the initial temperature was 1000 °C and increased to a final temperature of 1380 °C with a heating rate of 6.33 °C/min^28^. Furthermore, the effect of briquette thickness on iron recovery was also investigated^29^. In addition, the initial temperature may influence the iron recovery in the nuggets. Therefore, in this paper, we report the effect of different temperature patterns on the iron nugget formation and the recovery of iron in nuggets by varying the initial temperature.

## Methods

Titanomagnetite concentrate (TTM) was taken from one of the mining and beneficiation plants situated in Sukabumi, West Java, Indonesia. To remove the moisture content, the TTM was dried for 24 h in an oven at 130 °C. The TTM has a grain size of less than 0.212 mm (−65#), as listed in Table 1. The main minerals in the TTM, as determined by X-ray diffraction (XRD), were titanomagnetite (Fe_2.75_Ti_0.25_O_4_) with traces of ilmenite (FeTiO_3_). The chemical composition of TTM is given in Table 2 wherein the iron and titanium content were 55.35% and 6.70%, respectively. Coal as reductant was prepared to obtain a grain size of less than 35# (−0.425 mm) using a roll crusher, and a ball mill. The coal was dried for 24 h in an oven at 130 °C to remove its moisture content. Tables 3 and 4 listed the proximate and ultimate analyses of coal.

**Table 1:** Titanomagnetite grain size distribution (wt.%).

−325# (−0.044 mm)	−200# + 325# (0.044–0.074 mm)	−170# + 200# (0.074–0.088 mm)	−80# + 170# (0.088–0.177 mm)	+ 80# (+ 0.177 mm)
9.11	23.31	9.12	34.97	23.49

**Table 2:** Chemical composition of titanomagnetite concentrate (wt.%).

Fe_2.75_Ti_0.25_O_4_	FeTiO_3_	SiO_2_	MgO	Al_2_O_3_	MnO	P_2_O_5_	S	Other
78.16	8.31	2.82	1.86	3.55	0.50	0.08	0.005	4.71

**Table 3:** Coal proximate analysis (wt.%, adb).

Inherent moisture	Volatile matter	Ash	Fixed carbon
4.31	38.10	15.31	42.28

adb = air-dried basis.

**Table 4:** Coal ultimate analysis (wt.%, adb).

C	O	N	H	S
62.54	14.74	1.16	5.31	0.94

A mixture of TTM with 20 wt.% coal, where the weight of TTM was used as the basis, was formed into cylindrical briquettes with the help of a hydraulic press. Based on the preliminary experiments, the metal nuggets were formed if the coal was added to the mixture in the range of 20–30%^28^. Four grams of the mixture produced a briquette with a thickness of about 9 mm and a diameter of 15 mm. A coal layer was prepared at the bottom of a 20-mL alumina crucible equipped with a lid before a briquette was loaded into the crucible. More coal was added to the crucible after the briquette was loaded to support the reduction and maintain the reducing environment. A total of 5 g of coal was added for this purpose.

A muffle furnace without controlling the atmosphere was used for the experiments. The furnace temperature pattern followed an isothermal-temperature gradient profile. Three briquettes in separate crucible for each experimental parameter were prepared and reduced to obtain reliable results. After the experiment, the crucibles were removed from the muffle furnace and allowed to cool to room temperature. Then, the reduced briquettes were weighed and documented. Scanning electron microscopy (SEM) and energy-dispersive X-ray spectroscopy (EDS) were used to examine the reduced briquettes. Separation of the iron nuggets and the slag in the reduced briquettes was carried out manually by hand sorting with the help of tweezers. The nuggets and slag were weighed, and the size of the nuggets was measured by a caliper. The bisection of the nuggets was examined by optical microscopy and SEM–EDS. The slag was analyzed by XRD.

## Results and discussion

### Temperature pattern and physical appearance of reduced briquette

**Figure 1:**
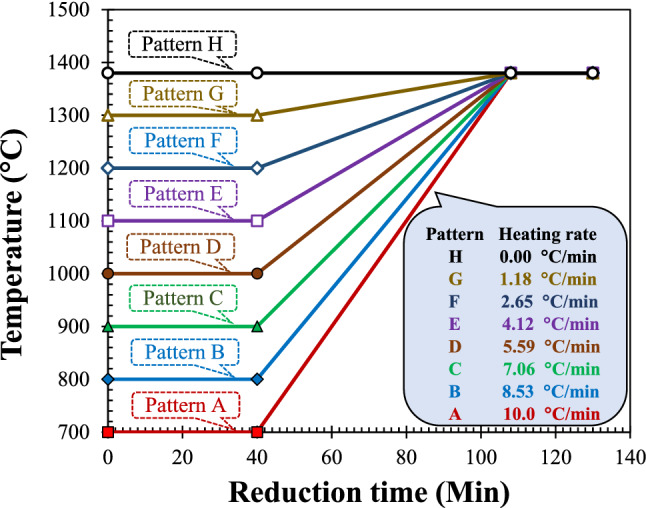
Temperature patterns.

As previously reported, iron nuggets were formed by reducing TTM/coal composite pellets under an isothermal-temperature gradient profile where the initial temperature was 1000 °C and the final temperature was 1380 °C^28^. An initial temperature of 1000 °C was chosen based on the results of previous experiments on the reduction of titanomagnetite by coal which showed that metallic iron was formed at temperatures higher than 900 °C^22^. Different initial temperatures may have an influence on nugget formation. Therefore, the initial temperature was varied, as shown in Fig. 1. Pattern A was started by setting the furnace temperature at 700 °C and held for 40 min at 700 °C, then the furnace temperature was increased to 1380 °C in 68 min with a heating rate of 10 °C/min and finally held at 1380 °C for 22 min. The total treatment time was 130 min. The same procedure was carried out on the other temperature patterns until the H pattern where the temperature was constant from start to the end at 1380 °C. For information, the temperature intended and set in the experiments was the furnace temperature, not the temperature at the surface of the briquette with coal in the alumina crucible.

**Figure 2:**
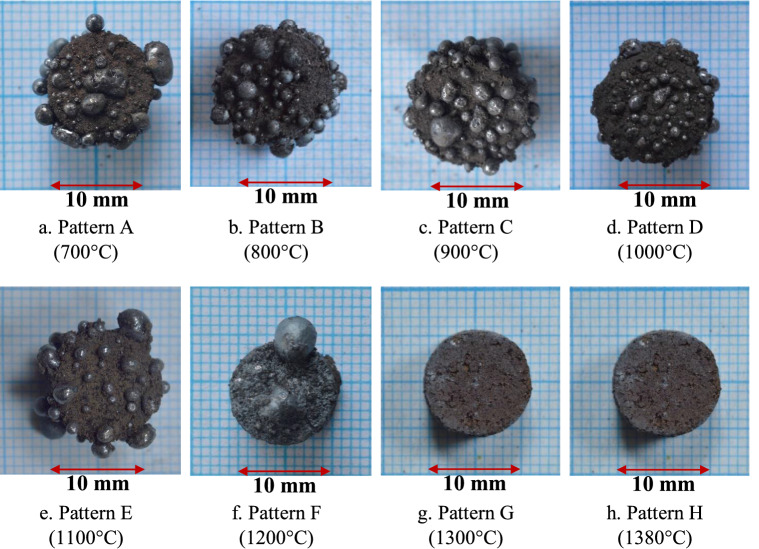
Reduced briquettes resulting from different temperature patterns.

Figure 2 shows the physical appearance of the reduced briquettes. The temperature pattern A to E formed iron nuggets in large numbers but with similar and smaller sizes. The F temperature pattern produced fewer nuggets, but they are larger in size. No nuggets were formed on reduced briquettes using the temperature patterns G and H. From the observation of this physical appearance, it can be concluded that the initial temperature of TTM/coal composite briquettes reduction under an isothermal-temperature gradient profile played a very important role in the formation of iron nuggets on the surface of reduced briquettes.

### Nugget size and iron recovery in Nugget

**Figure 3:**
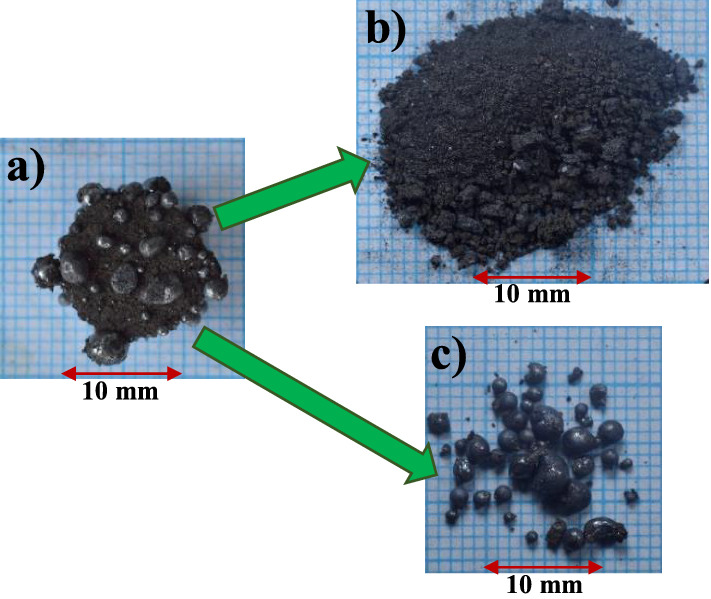
Separation of reduced briquette (**a**) into slag (**b**) and iron nuggets (**c**).

**Figure 4:**
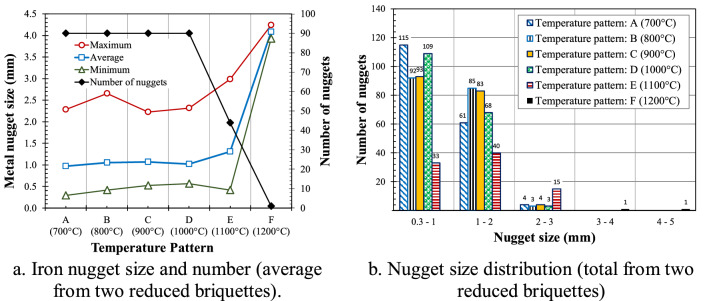
Iron nugget size and distribution for different temperature patterns.

The reduced briquettes were crushed using a mortar and the iron nuggets were separated from the slag as shown in Fig. 3. The results of the measurement of the nugget size are shown in Fig. 4a as a function of the temperature pattern from two reduced briquettes for each experimental parameter. The temperature patterns G and H were not plotted in the graph because no nuggets were visible on the surface of reduced briquettes. The number of nuggets is also shown in the Fig. 4a. It can be seen that the maximum nugget size of about 4 mm can be achieved using the F temperature pattern. In addition, it is also seen that only one large nugget was produced using the F temperature pattern. In the A to D temperature patterns, the average size of the nuggets was about 1 mm with a maximum size ranging from 2.2 to 2.7 mm. Approximately 90 nuggets were separated from each reduced briquette using A to D temperature patterns. These nuggets are larger than 0.3 mm. Nuggets smaller than 0.3 mm remained in the slag which can be further recovered by using for example a magnetic separator. The temperature pattern E showed a maximum nugget size of 3 mm with an average size of 1.3 mm. The size of nuggets from pattern E was between the sizes of nuggets produced from temperature patterns D and F. The number of nuggets from one reduced briquette using temperature pattern E was 44. Details of the size distribution of nugget resulting from each temperature pattern are shown in Fig. 4b.

**Figure 5:**
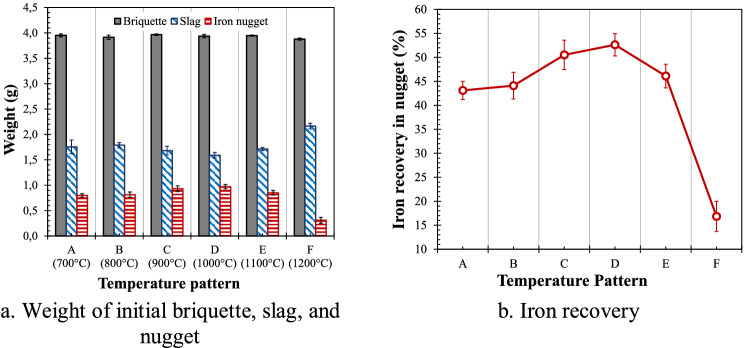
Weight of iron nugget, slag, and iron recovery for different temperature patterns (average from three reduced briquettes).

The weight of the reduced briquettes, slag, and iron nuggets is shown in Fig. 5a. Based on these data, the iron recovery in the nuggets was calculated where the results are depicted in Fig. 5b. It can be seen that iron recovery in nuggets increased from temperature pattern A with an initial temperature of 700 °C and achieved optimum iron recovery in temperature pattern D with an initial temperature of 1000 °C. Subsequently, the iron recovery decreased as shown in temperature patterns E and F with initial temperatures of 1100 °C and 1200 °C, respectively.

As previously reported^22^ for solid-state reduction of TTM under isothermal conditions, at 700 or 800 °C, a certain amount of magnetite in titanomagnetite was reduced to wustite but no metallic iron was formed. Although no metallic iron was formed at 700 or 800 °C in the first initial stages of temperature patterns A and B, further reduction of the iron oxides in the briquettes occurred in the second stage, where heating was carried out from 700 or 800 °C to 1380 °C resulting in iron nuggets as shown clearly on the surface of the reduced briquettes in Fig. 2a,b. The iron recovery in the nuggets for temperature patterns A and B was similar at about 43% (Fig. 5b).

As the initial temperature increased to 900 °C, metallic iron was formed with a metallization degree of about 13%^22^ attainable during the first isothermal step for a duration of 40 min. Therefore, the iron recovery in nuggets increased from 43% (temperature pattern B) to 50% (temperature pattern C). At 1000 °C, the metallization degree increased to about 30%^22^ during the isothermal step so that the recovery increased to 53% for the nuggets resulting from the temperature pattern D. Temperature pattern D with an initial temperature of 1000 °C seems to be the optimum temperature pattern for achieving high iron recovery in the form of nuggets.

Further increase in initial temperature tended to reduce the iron recovery in the nuggets as well as the nugget formation. At an initial temperature of 1100 °C, the metallization degree increased to about 50%^22^ at the first isothermal step for 40 min, but the iron recovery in the nuggets decreased. This can be caused by the formation of large amounts of metallic iron on the surface of the briquette at the first stage of isothermal which can inhibit the migration of metal from the center to the outer surface of the briquette. The results of the previous study^28,29^ indicated that the important step for nugget formation was the second stage on the isothermal–temperature gradient profile where the temperature was increased with a certain heating rate towards 1380 °C.

Further increasing the initial temperature to 1200 °C reduced the porosity on the briquette surface in addition to increasing the metallization degree so that the formation of nuggets became more difficult. At initial temperatures of 1300 °C and 1380 °C, no nugget was formed although the metallization degree throughout the briquettes increased. Besides the initial temperature, the heating rate can also affect the formation of iron nuggets.

Geng et al.^17^ investigated the reduction of TTM pellets by embedding in coal as a reducing agent under isothermal conditions in the temperature range of 1100–1300 °C. The reduced pellets were crushed and ground to less than 0.043 mm and the metallic iron was recovered by magnetic separator technique. It was reported that the optimum temperature was 1200 °C to achieve high iron recovery and high iron grade. In the temperature range of 1250 to 1300 °C, the semi molten phase was formed which prevented the reducing gas from the embedded coal entering the center of the pellet^17,21^.

**Figure 6:**
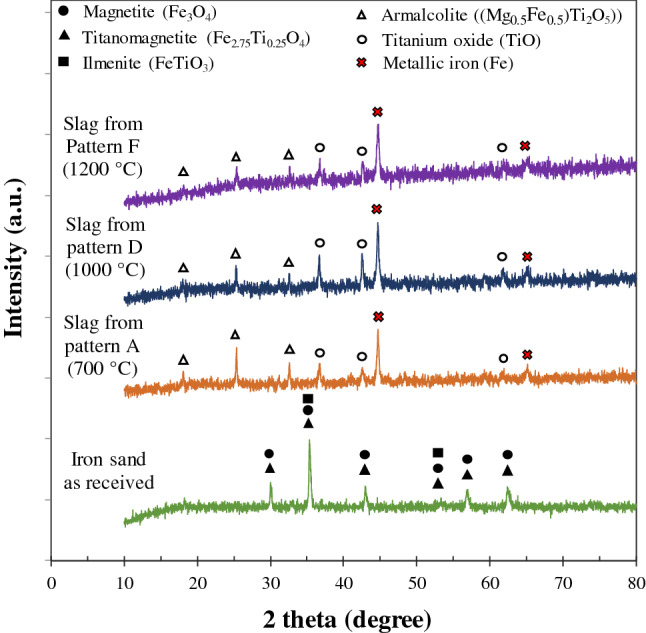
XRD pattern of slag after iron nugget separation.

As mentioned earlier, the nuggets which were recovered have a size larger than 0.3 mm. The nuggets that were less than 0.3 mm in size cannot be recovered by hand sorting where the other techniques must be applied. The slag was analyzed by XRD where the results are shown in Fig. 6 for temperature patterns A, D, and F. It can be seen that the metallic iron was still dominant in the slag. Titanium was presented as titanium oxide (TiO) and armalcolite ((Mg_0.5_Fe_0.5_)Ti_2_O_5_).

The Indonesian TTM was used as experimental material by previous researchers. Gao et al.^13^ reported that the phases in a TTM mixture with 25% coal reduced at 1250 °C were metallic iron, ilmenite, and armalcolite. A similar experiment was carried out by Geng et al.^17^ and found that the phases in pellets reduced by embedding in the coal were metallic iron, ilmenite, and pseudobrookite (FeTi_2_O_5_). Recently, Zhao et al.^21,23^ reported that the phases in the reduced mixture consisting of TTM and 25% coal at 1200–1300 °C were metallic iron, ilmenite, and anosovite ((Fe,Mg)Ti_2_O_5_). From the experiments mentioned above, the iron nuggets were not formed, and the metallic iron was still attached to other oxides. Therefore, a series of crushing and grinding treatments are required to liberate the metallic iron.

### Microstructure and SEM–EDS observation

**Figure 7:**
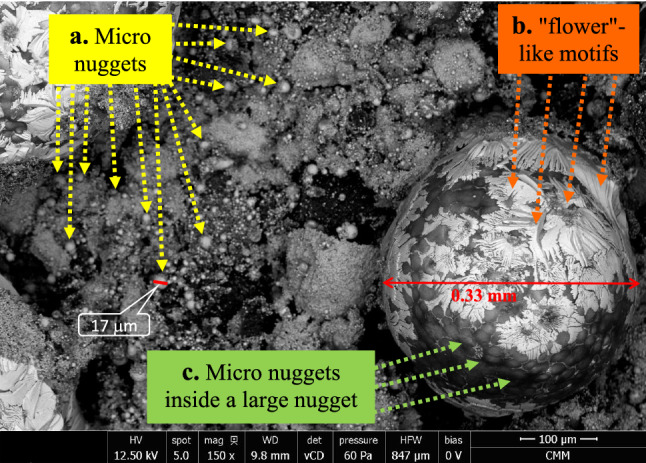
SEM image on the surface of the reduced briquette produced using the temperature pattern A.

**Figure 8:**
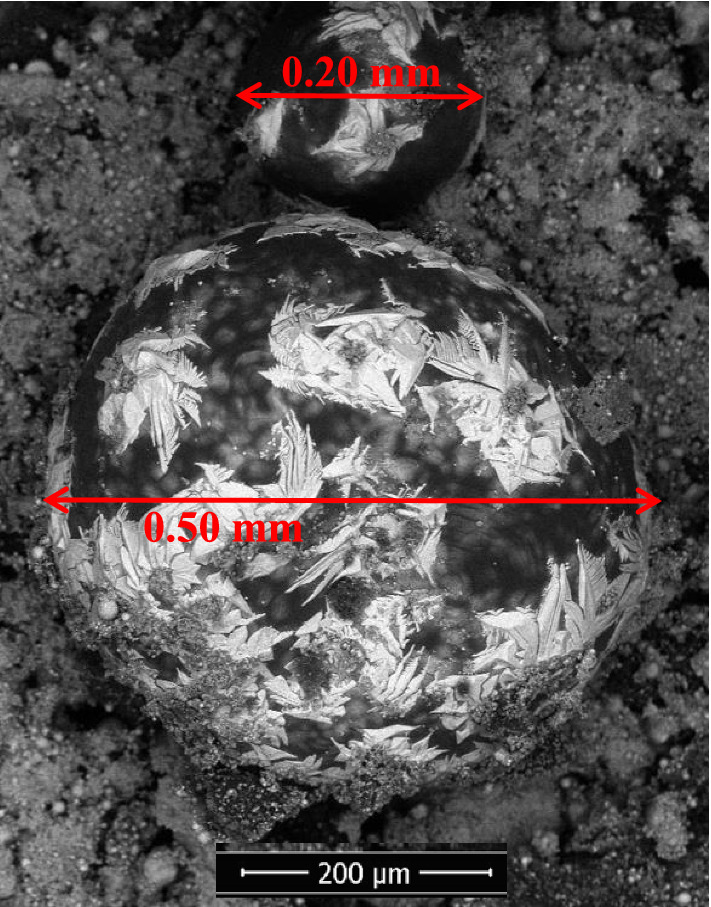
SEM image on the surface of the nugget produced using the temperature pattern A.

The surface of the briquette reduced under temperature pattern A was analyzed by SEM–EDS, as shown in Fig. 7. Many nuggets with a size of less than 20 μm, called micro nuggets, were visible on the surface of the briquette. These micro nuggets have not been agglomerated to form larger nuggets. These micro nuggets were impossible to separate from the slag by hand sorting and remained in the slag as shown by the XRD pattern in Fig. 6, where the metallic iron was the dominant phase. Another separation technique is required, e.g., a magnetic separator to recover these micro nuggets. In addition, a nugget with a size of 0.33 mm was clearly visible on the surface of the briquette. This nugget size may still be difficult to separate from the slag manually by hand sorting as shown in Fig. 4a for the minimum size of the nuggets that was counted for iron recovery. On the surface of this nugget, “flower”-like motifs with light gray color revealed. Inside this nugget, the shape like micro nuggets can be seen clearly where the micro nuggets were covered by a transparent layer. A similar phenomenon was observed in other nuggets as shown in Fig. 8 where the agglomerated micro nuggets were covered by a transparent layer forming a large nugget size.

**Figure 9:**
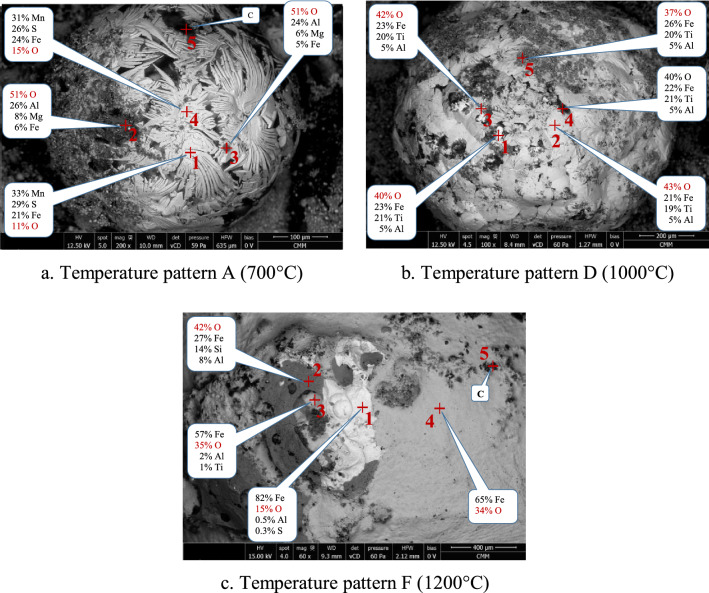
SEM–EDS on the surface of reduced nuggets.

SEM–EDS analysis on the surface of nuggets which were produced from temperature patterns A, D, and F are shown in Fig. 9. From Fig. 9a, it is clear that the formation of “flower”-like motifs on the surface of nuggets in Fig. 7 was caused by the presence of manganese together with sulfur and iron forming a sulfide phase that had a lower melting point. Manganese came from TTM as listed in Table 2 and sulfur from coal as the sulfur content in the TTM is very low (0.005%). The combination of iron, manganese, and sulfur may form FeS⋅2MnS with a melting point of 1128 °C that solidified last on the surface of nuggets. The other phases are oxides containing aluminum, magnesium, and iron. Figure 9b,c show the oxides formed on the nugget’s surface containing iron, titanium, aluminum, and oxygen.

**Figure 10:**
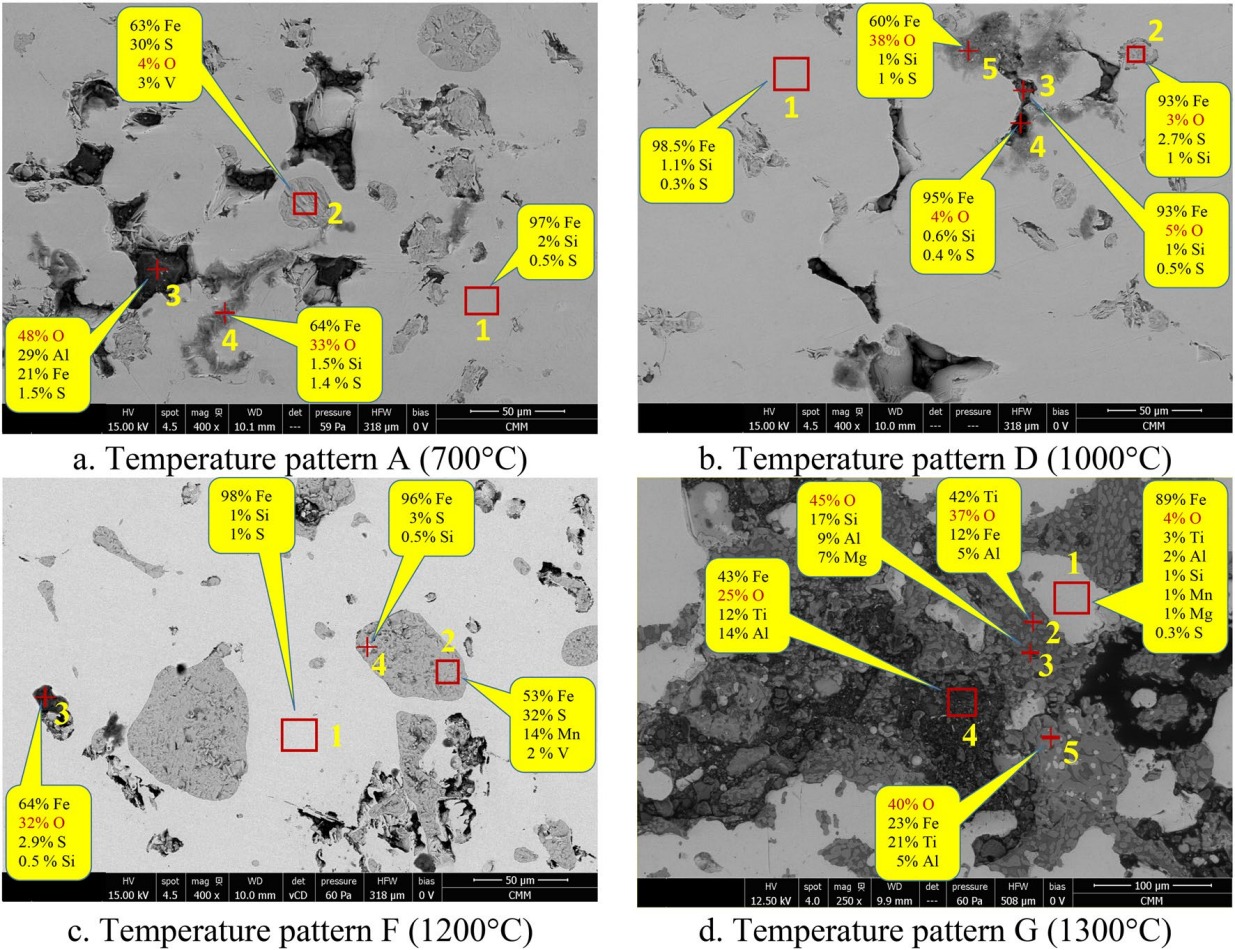
SEM–EDS of the bisection of iron nugget (**a**–**c**) and on the polished surface of reduced briquette (**d**).

Even though on the surface of the nuggets the dominant phases were oxides and sulfides, on the inside of the nugget the dominant phase was metallic iron, as shown in Fig. 10a–c for temperature patterns A, D, and F. In area 1 in Fig. 10a, the iron content was 97% with 2% silicon and 0.5% sulfur as impurities based on EDS analysis. Similar compositions were observed in area 1 in Fig. 10b,c which were produced from temperature patterns D and F. The presence of carbon was not considered due to the accuracy of the measurements. Hu et al.^22^ reported that the carbon content was 1.51% in the nuggets produced by reduction of TTM by coal at 1350 °C.

In area 2 of Fig. 10a, inclusions consisting of 63% of iron and 30% of sulfur were observed. The combination of iron and sulfur in that composition formed troilite (FeS), which has a melting point of 1194 °C. At point 3 in Fig. 10a, there are oxides that can be trapped in the nuggets during the agglomeration process of micro nuggets into larger nuggets as shown in Figs. 7 and 8. Iron oxides were also found in the nuggets as shown at point 4 in Fig. 10a. Oxides have higher melting points; therefore, voids can be found around the oxides which may have formed during the solidification of the nuggets. A similar phenomenon was observed in the nugget resulted from the reduced briquette using the temperature patterns D and F, as shown in Fig. 10b, c, respectively. In general, the area on the cross-section of the nugget can be divided into three, namely the matrix as metallic iron, precipitations or inclusions in the form of (Fe,Mn)_x_S_y_ and oxides or voids.

Using the temperature pattern G, no nuggets were formed; therefore, the SEM–EDS analysis was carried out on the polished surface of the reduced briquette, as shown in Fig. 10d. The light gray area (area 1) in Fig. 10d is metallic iron containing small amounts of oxygen, titanium, aluminum, silicon, manganese, magnesium, and sulfur. The aluminum, magnesium, and titanium are present as oxides. In the other dark gray areas, iron was presented as oxides along with titanium, aluminum, and magnesium oxides. At this pattern, the initial temperature was 1300 °C, where the metallic iron was formed instantly on the briquette surface and created a crust that hindered the formation of iron nuggets.

**Figure 11:**
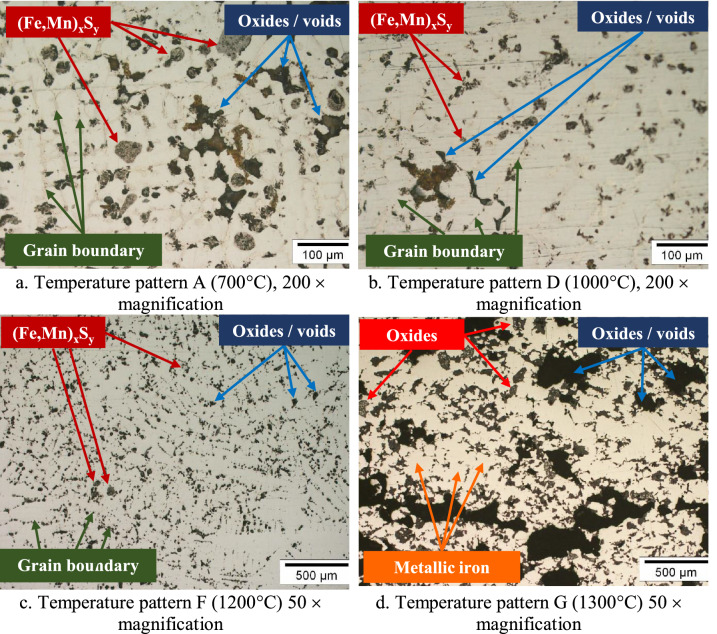
Optical microscopy image of the bisection of iron nugget (**a**–**c**) and polished surface of reduced briquette (**d**).

The microstructure images from optical microscopy of the bisection of nuggets resulting from temperature patterns A, D, and F are shown in Fig. 11a–c while of the polished surface of the reduced briquette is shown in Fig. 11d. Grain boundaries are clearly visible in Fig. 11a with a magnification of 200 × , which may be formed from the agglomeration of small metal particles with one another. It was believed that the metal was not completely melted where the metal phase was semi liquid. During agglomeration, the unreduced oxides were trapped between one metal particle and another. Grain boundaries and trapped oxides, as well as sulfide precipitations at the grain boundaries, can be seen clearly in Fig. 11c. Figure 11d shows metallic iron formed on the surface of the reduced briquettes. To recover the metallic iron with a magnetic separator, a comminution step consisting of crushing and grinding is required to liberate the metallic iron particles from the unreduced oxides. This technique was reported by pervious researchers^13,17,21,23^.

From the phenomena above and previous works^22,28,29^, the mechanism of nugget formation started with the reduction of iron oxide in the TTM through CO gas from carbon in the coal to form metallic iron at a temperature of more than 800 °C. Then, the metallic iron formed spherical particles or grew like whiskers during the heating stage to 1380 °C. The presence of sulfur lowered the iron’s melting point, which can initiate nucleation sites of metallic iron particles. As the temperature reached 1380 °C, the micro iron nuggets were formed due to the migration of small metallic iron particles in the form of spherical or whiskers, interconnected from one to another and agglomerated to form a larger globular (nugget). As the temperature was held at 1380 °C, the size of the iron nuggets became larger.

## Conclusion

The effect of temperature patterns was studied on the iron nugget formation during the reduction of titanomagnetite concentrate by coal. The initial temperatures were varied from 700 to 1380 °C while the final temperature was kept constant at 1380 °C. The initial temperatures of 700–1100 °C produced many iron nuggets up to 3 mm in size and an initial temperature of 1200 °C produced one iron nugget with a size of about 4 mm. The mechanism of iron nugget formation started with the reduction of iron oxide in the TTM to form metallic iron at a temperature of more than 800 °C, the metallic iron formed spherical particles during the heating stage to 1380 °C. As the temperature reached 1380 °C, the iron nuggets were formed due to the migration of small metallic iron particles, interconnected from one to another and agglomerated to form larger iron nuggets. Initial temperatures of 1300 and 1380 °C did not produce any iron nuggets due to the formation of metallic iron crust on the surface of the reduced briquettes. The optimum initial temperature was 1000 °C to achieve high iron recovery in the nuggets.

## Data Availability

All data generated or analysed during this study are included in this published article.

## References

[1] Ure, C. R. Alternative ironmaking at BHP New Zealand Steel. *Proc. 2000 Electric Furnace Conference*, AIST, Warrendale, USA, 535–546, http://digital.library.aist.org/pages/PR-324-067.htm (2000).

[2] Steinberg WS, Geyser W, Nell J (2011). The history and development of the pyrometallurgical processes at Evraz Highveld Steel & Vanadium. J. South. Afr. Inst. Min. Metall..

[3] Geldenhuys, I. J., Akdogan, G. & Reynolds, Q. G. Towards sustainable processing of vanadium-bearing titaniferous magnetite deposits - an overview of barriers and opportunities. *IMPC 2020*, Cape Town, South Africa, 2566–2581, http://www.mintek.co.za/Pyromet/Files/2021Geldenhuys.pdf (2020).

[4] Pang Z, Lv X, Jiang Y, Ling J, Yan Z (2020). Blast furnace ironmaking process with super-high TiO_2_ in the slag: Viscosity and melting properties of the slag. Metal. Mater. Trans. B..

[5] Fu WG, Xie HE (2011). Progress in technologies of vanadium-bearing titanomagnetite smelting in PanGang. Steel Res. Int..

[6] Wang S (2017). Reduction behaviors of iron, vanadium and titanium oxides in smelting of vanadium titanomagnetite metallized pellets. JOM.

[7] Chen D (2011). Solid state reduction of Panzhihua titanomagnetite concentrates with pulverized coal. Min. Eng..

[8] Hu T, Lv X, Bai C, Lun Z, Qiu G (2013). Reduction behavior of Panzhihua titanomagnetite concentrates with coal. Metal. Mater. Trans. B..

[9] Sun H, Dong X, She X, Xue Q, Wang J (2013). Solid state reduction of titanomagnetite concentrate by graphite. ISIJ Int..

[10] Geng C (2015). Effect of Na_2_SO_4_ on the embedding direct reduction of beach titanomagnetite and the separation of titanium and iron by magnetic separation. ISIJ Int..

[11] Zhao W (2017). Investigation of reduction mechanism and kinetics of vanadium titanomagnetite carbon composite hot briquette at 1173–1373 K. Steel Res. Int..

[12] Hu T, Lv X, Bai C (2016). Enhanced reduction of coal-containing titanomagnetite concentrates briquette with multiple layers in rotary hearth furnace. Steel Res. Int..

[13] Gao E, Sun T, Liu Z, Geng C, Xu C (2016). Effect of sodium sulfate on direct reduction of beach titanomagnetite for separation of iron and titanium. J. Iron. Steel Res. Int..

[14] Liu Y, Zhang J, Liu Z, Xing X (2016). Phase transformation behavior of titanium during carbothermic reduction of titanomagnetite ironsand. Int. J. Miner. Metal. Mater..

[15] Wang M (2016). Recovery of iron from chromium vanadium-bearing titanomagnetite concentrate by direct reduction. JOM.

[16] Zhang Y (2017). A novel process for the recovery of iron, titanium, and vanadium from vanadium-bearing titanomagnetite: sodium modification–direct reduction coupled process. Int. J. Miner. Metal. Mater..

[17] Geng C, Sun T, Ma Y, Xu C, Yang H (2017). Effects of embedding direct reduction followed by magnetic separation on recovering titanium and iron of beach titanomagnetite concentrate. J. Iron. Steel Res. Int..

[18] Zhang Y (2018). A method for recovery of iron, titanium, and vanadium from vanadium-bearing titanomagnetite. Int. J. Miner. Metal. Mater..

[19] Sarkar BK, Kumar N, Dey R, Das GC (2018). Optimization of quenching parameters for the reduction of titaniferous magnetite ore by lean grade coal using the Taguchi method and its isothermal kinetic study. Metal. Mater. Trans. B..

[20] Smirnov KI, Salikhov SP, Roshchin VE (2019). Solid-phase reduction of iron from Suroyam titanomagnetite ore during metallization in rotary kiln. Mater. Sci. Forum..

[21] Zhao Y, Sun T, Zhao H, Chen C, Wang X (2019). Effect of reductant type on the embedding direct reduction of beach titanomagnetite concentrate. Int. J. Miner. Metal. Mater..

[22] Zulhan Z, Adhiwiguna IS, Fuadi A, Saleh N (2021). Solid-state reduction of an Indonesian iron sand concentrate using subbituminous coal. Can. Metall. Q..

[23] Zhao Y, Sun T, Wang Z (2021). Extraction of iron from refractory titanomagnetite by reduction roasting and magnetic Separation. ISIJ Int..

[24] Matsumura T (1998). Direct production of molten iron from carbon composite iron ore pellet. La Revue de Métallurgie CIT..

[25] Kobayashi, I., Tanigaki, Y. & Uragami, A. A new process to produce iron directly from fine iron ore and coal. *60th Ironmaking Conference*, Baltimore, USA, 649–657, http://digital.library.aist.org/pages/PR-016-54.htm (2001).

[26] Harada T, Tsuge O, Kobayashi I, Tanaka H, Uemura H (2005). The development of new iron making processes. Kobelco Tech. Rev..

[27] Kikuchi S, Ito S, Kobayashi S, Tsuge O, Tokuda H (2010). ITmk3 process. Kobelco Tech Rev..

[28] Zulhan Z, Lo F (2021). Iron nugget formation from iron sand/coal composite pellets under isothermal-temperature gradient profiles. Ironmak. Steelmak..

[29] Zulhan Z, Husnaa Z, Basuki EA (2022). Effect of briquette thickness on iron nugget formation in fluxless processing of iron sand concentrate under isothermal – temperature gradient profiles. ISIJ Int..

